# Antimicrobial resistance of *Campylobacter* isolates from small scale and backyard chicken in Kenya

**DOI:** 10.1186/s13099-016-0121-5

**Published:** 2016-08-27

**Authors:** Tuan Ngoc Minh Nguyen, Helmut Hotzel, John Njeru, Joyce Mwituria, Hosny El-Adawy, Herbert Tomaso, Heinrich Neubauer, Hafez M. Hafez

**Affiliations:** 1 Hung Vuong University, Phu Tho, Vietnam; 2 Friedrich-Loeffler-Institut, Institute of Bacterial Infections and Zoonoses, Jena, Germany; 3 Institute of Poultry Diseases, Free University Berlin, Berlin, Germany; 4 Centre for Microbiology Research (CMR), Kenya Medical Research Institute, Nairobi, Kenya; 5 Department of Poultry Diseases, Faculty of Veterinary Medicine, Kafrelsheikh University, Kafrelsheikh, Egypt

**Keywords:** *Campylobacter*, Antibiotic resistance, Microdilution, Chicken, Kenya

## Abstract

**Background:**

Thermophilic *Campylobacter* species are a major cause of bacterial foodborne diarrhoea in humans worldwide. Poultry and their products are the predominant source for human campylobacteriosis. Resistance of *Campylobacter* to antibiotics is increasing worldwide, but little is known about the antibiotic resistance in *Campylobacter* isolated from chicken in Kenya. In this study, 35 suspected *Campylobacter* strains isolated from faeces and cloacal swabs of chicken were tested for their susceptibility to seven antibiotics using a broth microdilution assay and molecular biological investigations.

**Results:**

Overall, DNA of thermophilic *Campylobacter* was identified in 53 samples by PCR (34 *C. jejuni*, 18 *C. coli* and one mix of both species) but only 35 *Campylobacter* isolates (31 *C. jejuni* and 4 *C. coli*) could be re-cultivated after transportation to Germany. Isolates were tested for their susceptibility to antibiotics using a broth microdilution assay. Additionally, molecular biological detection of antibiotic resistance genes was carried out. *C. jejuni* isolates showed a high rate of resistance to nalidixic acid, tetracycline and ciprofloxacin of 77.4, 71.0 and 71.0 %, respectively. Low resistance (25.8 %) was detected for gentamicin and chloramphenicol. Multidrug resistance in *C. jejuni* could be detected in 19 (61.3 %) isolates. Resistance pattern of *C. coli* isolates was comparable. Resistance to ciprofloxacin was confirmed by MAMA–PCR and PCR–RFLP in all phenotypically resistant isolates. The *tet*(O) gene was detected only in 54.5 % of tetracycline resistant *C. jejuni* isolates. The *tet*(A) gene, which is also responsible for tetracycline resistance, was found in 90.3 % of *C. jejuni* and in all *C. coli* isolates. Thirteen phenotypically erythromycin-resistant isolates could not be characterised by using PCR–RFLP and MAMA–PCR.

**Conclusions:**

To the best of our knowledge, this study is the first report about resistance to antibiotics in thermophilic *Campylobacter* originating from chicken in Kenya. *Campylobacter* spp. show a high level of resistance to ciprofloxacin, nalidixic acid and tetracycline but also a remarkable one to chloramphenicol and gentamicin and they are multidrug resistant. Resistance to antibiotics is a global public health concern. In Kenya, resistance surveillance needs further attention in the future. Efforts to establish at least a National Laboratory with facilities for performing phenotypic and genotypic characterization of thermophilic *Campylobacter* is highly recommended.

## Background

Thermophilic *Campylobacter* (*C.*) species have become the most frequent cause of bacterial gastroenteritis in humans worldwide [1]. Campylobacteriosis exceed the total number of those caused by *Salmonella, Shigella*, and *Escherichia coli* O157:H7 in humans, recently [2]. *Campylobacter* infections are normally self-limiting in adults but can cause diarrhoea or even mortality in children in developing as well as in developed countries [3, 4].

A study from western Kenya showed that 20 % of patients with diarrhoea were infected by *Campylobacter* but in the group of children below 5 years *Campylobacter* was represented with 42 % [5].

Commercial poultry and free-living birds are natural reservoirs of thermophilic campylobacters. The organism has been isolated from numerous bird species, including *Columbiformes* and domestic and free-living *Galliformes* and *Anseriformes*. *C. jejuni* has been found in all areas of commercial poultry production [6, 7]. Prevalence rates in poultry, especially in slaughter-age broiler flocks, could reach 100 % on some farms. Although, *Campylobacter* is insignificant for poultry health, it is a predominant cause of foodborne gastroenteritis in humans worldwide, and contaminated poultry meat is recognized as the main source of human infections [7, 8]. In general, the knowledge about *Campylobacter* in Kenya is limited. Most of the published reports describe prevalence and antibiotic resistance in *Campylobacter* of human origin [4, 9–11]. Other reports gave information about *Campylobacter* as cause of foodborne diseases [12] and contamination of raw chicken and beef from butcheries and markets in Nairobi [13]. Information on thermophilic *Campylobacter* of animal origin from Kenya is lacking.

Resistance against antibiotics in bacteria is of public health concern. Most commonly used drugs in treatment of campylobacteriosis in humans are erythromycin, fluoroquinolones or tetracycline [14]. Although, this antimicrobial treatment is usually not necessary, however the misuse of antibiotics is widespread in Kenya [5]. Attention on resistance of *Campylobacter* is raising and warning has been launched not to misuse antibiotics such as macrolides, fluoroquinolones or alternative drugs [15]. Kenyan *Campylobacter* isolates from humans showed a high resistance rate against erythromycin (52 %), but only low resistance to ciprofloxacin, tetracycline and nalidixic acid with 6, 18 and 26 % in the past, respectively [5].

Clinical breakpoints of *Campylobacter* susceptibility based on epidemiological cut-off (ECOFF) values were recommended. EUCAST MIC distributions incorporate human and veterinary clinical data from several sources worldwide [16]. The method of choice for testing antibiotic susceptibility and determination of minimum inhibitory concentration (MIC) values of *Campylobacter* isolates is the broth microdilution assay [17, 18].

In addition to phenotypical determination of antibiotic resistance, genetic analysis of resistance determinants in *Campylobacter* can be carried out. A replacement of threonine by isoleucine at amino acid 86 in the *gyr*A gene [19, 20] and a mutation at position 2074 and 2075 on the 23S rRNA gene are the main mechanisms for fluoroquinolone and erythromycin resistance, respectively [21]. Presence of *tet*(O) and/or *tet*(A) genes is responsible for tetracycline resistance [22]. A mismatch amplification mutation assay (MAMA–PCR) can be used for detection of the mutations in *gyr*A and 23S rRNA genes in *C. coli* and *C. jejuni* responsible for ciprofloxacin and erythromycin resistance, respectively [21, 23]. PCR-restriction fragment length polymorphism (PCR–RFLP) technique [24] is available for detection of erythromycin resistance as well as specific PCR assays for *tet*(O) and *tet*(A) genes. These methods allow the investigation of antibiotic resistance of *Campylobacter* even in samples from which no *Campylobacter* could be isolated.

To the best of our knowledge there is no report available about antibiotic resistance of thermophilic *Campylobacter* species isolated from chicken in Kenya. MICs and results of molecular assays on the resistance of recent Kenyan *C. coli* and *C. jejuni* are presented.

## Methods

### Sample collection and *Campylobacter* isolation

In total, 35 geographically different native breed layer flocks were sampled. The chickens were housed in backyards and homesteads of small scale farmers from the outskirts of Thika, a town 40 km northeastern of Nairobi, Kenya. Farmers kept between 10 and 1000 layers. The birds were fed on commercially formulated ration from different sources and sometimes supplied with the leftover and residual food. All the manufactures used antibiotics as part of the ingredients in the feed. During the rearing of these chickens, antibiotics were used for prevention and treatment of diseases without any instructions. Ten to 30 cloacal swabs and faecal samples were collected from each flock according to flock size. *Campylobacter* were isolated in Kenya Medical Research Institute, Nairobi according to the guidelines of ISO 10272-1 [25]. The isolates were preserved in 1.5 ml Eppendorf tubes filled with skimmed milk medium for 1-week transportation from Kenya to Friedrich–Loeffler-Institut, Jena, Germany for further laboratory analysis. *Campylobacter* strains were re-cultivated on both Mueller–Hinton agar and CCDA (Oxoid GmbH, Wesel, Germany) under microaerophilic conditions (5 % O_2_, 10 % CO_2_, and 85 % N_2_) at 37 °C for 48–72 h.

### DNA extraction

DNA from viable bacteria was extracted using the High Pure PCR Template Preparation Kit™ (Roche Diagnostics, Mannheim, Germany) according to the manufacturer’s instructions. Skimmed milk samples of *Campylobacter* that could not be re-cultivated were treated with phenol–chloroform to extract DNA. Briefly, 500 µl of skimmed milk medium was boiled for 5 min. After cooling the liquid was mixed with 500 µl buffer-saturated phenol (Carl Roth GmbH, Karlsruhe, Germany) and centrifuged for 5 min. at 13,400 rpm (miniSpin, Eppendorf, Hamburg, Germany). 500 µl chloroform/isoamyl alcohol (24:1 vol/vol) was added to the aqueous phase, mixed and centrifuged for 5 min. at 13,400 rpm. DNA from the aqueous phase was precipitated by mixing with 0.6 volume of isopropanol at room temperature. After centrifugation, the supernatant was discarded and the DNA was air dried and finally dissolved in 50 µl 10 mM Tris (Carl Roth GmbH).

### Multiplex PCR for identification of *Campylobacter* species

A mPCR assay was used to identify thermophilic *Campylobacter* species (*C. jejuni*, *C. coli*, and *C. lari*) as described by El-Adawy et al. [26]. Briefly, the PCR was performed in a 50-μl reaction mixture containing 5.0 μl of 10 × *Taq* reaction buffer complete (Jena Bioscience GmbH, Jena, Germany), 2.0 μl of dNTP mix (2 mM each; Carl Roth GmbH), 2.0 μl of each primer (Jena Bioscience GmbH), and 0.2 μl of *Taq* Pol thermostable DNA polymerase (Jena Bioscience GmbH). Amplification reactions were carried out in a TRIO Thermoblock cycler (Biometra, Göttingen, Germany) using the following programme: one cycle of 1 min at 96 °C was followed by 35 cycles each consisting of 60 s at 95 °C of denaturation, 90 s at 59 °C of annealing, and 60 s at 72 °C of elongation. The PCR was terminated after a final extension step of 5 min. at 72 °C. Amplification generated 857, 589, 522, and 462 base pair DNA fragments specific for the genus *Campylobacter* and the species *C. jejuni*, *C. lari*, and *C. coli*, respectively. For analysis, 20 μl of PCR products were subjected to electrophoresis in a 1.5 % agarose gel for 1 h, stained with ethidium bromide (0.5 μg/ml), and visualized under UV light. Results were documented using BioImage system GeneGenius (Syngene, Synoptics Ltd., Cambridge, UK). Reference strains *C. jejuni* DSM 4688, *C. coli* DSM 4689, and *C. lari* DSM 11375 (Deutsche Sammlung von Mikroorganismen und Zellkulturen GmbH, Braunschweig, Germany) were used as positive controls.

### Antimicrobial susceptibility testing and determination of MICs

The antimicrobial susceptibility of *C. jejuni* and *C. coli* isolates was tested against seven antibiotic agents (chloramphenicol, erythromycin, ciprofloxacin, nalidixic acid, gentamicin, streptomycin, and tetracycline) using the Sensititre™ Campylobacter plates—EUCAMP (Trek Diagnostic Systems Ltd., East Grinstead, UK). The MIC values were detected using different concentration ranges as previously described [23]. Briefly, *Campylobacter* isolates grown on Mueller–Hinton agar (Oxoid GmbH) supplemented with 10 % bovine blood under microaerophilic conditions were suspended in NaCl solution (0.9 %) to obtain a turbidity corresponding to a McFarland standard of 0.5 (Dr. Lange, CADAS photometer 30, Berlin, Germany). One-hundred and fifty milliliters of the above suspension were diluted with 10 ml Mueller–Hinton broth (Oxoid GmbH) resulting in a concentration of approximately 10^6^–10^7^ colony forming units (cfu)/ml. One hundred milliliters of the inoculum was filled in each well of the plate; the plates were sealed and incubated at 37 °C for 24 h under microaerophilic conditions. Results were read either visually or photometrically (Tecan Deutschland GmbH, Crailsheim, Germany) using the computer program easyWIN fitting (version V6.1, 2000). *C. jejuni* DSM 4688 and *C. coli* DSM 4689 (Deutsche Sammlung von Mikroorganismen und Zellkulturen GmbH) were included in each batch of broth microdilution assay for quality control. The lowest concentration of antibiotics that prevents visible growth of the microorganism is defined as the MIC.

### Molecular biological detection of antibiotic resistance determinants

Extracted *Campylobacter* DNA from all samples and strains was used for molecular biological determination of selected antibiotic resistance determinants by PCR.

### Erythromycin resistance

Detection of mutations at positions 2074 and 2075 in domain V of the 23S rRNA gene, which mediates resistance to erythromycin, was carried out by MAMA–PCR and PCR–RFLP as described previously [21, 24]. Genes responsible for resistance of erythromycin and ciprofloxacin were tested at two loci using MAMA–PCR and PCR–RFLP. Primers and their sequences are given in Table 1.

**Table 1:** List of primers and primer sequences used for detection of antimicrobial resistance genes

Antibiotic	Method	Primer	Sequence (5’–3’)	Amplicon length (bp)	Reference
Erythromycin	MAMA–PCR^a^	23SRNA-F ERY2075-R ERY2074-R	TTA GCT AAT GTT GCC CGT ACC G TAG TAA AGG TCC ACG GGG TCG C AGT AAA GGT CCA CGG GGT CTG G	485	[17]
	PCR–RFLP^a^	F2-campy-23S R2-campy-23S	AAT TGA TGG GGT TAG CAT TAG C CAA CAA TGG CTC ATA TAC AAC TGG	316	[20]
Ciprofloxacin	MAMA–PCR^b^	CampyMAMAgyrA1 CampyMAMAgyrA5	TTT TTA GCA AAG ATT CTG AT CAA AGC ATC ATA AAC TGC AA	265	[19]
	MAMA–PCR^c^	GZgyrACcoli3F CanpyMAMAgyrA8	TAT GAG CGA TAT TAT CGG TC TAA GGC ATC GTA AAC AGC CA	192	[24]
Tetracycline	*tet*(O) PCR	DMT 1 DMT 2	GGC GTT TTG TTT ATG TGC G ATG GAC AAC CCG ACA GAA GC	559	[25]
	*tet*(A) PCR	Tet(A)-F Tet(A)-R	GTG AAA CCC AAC ATA CCC C GAA GGC AAG CAG GAT GTA G	888	[18]
	*tet*(A) PCR	tet-A-1 tet-A-2	GCT CAC GTT GAC GCA GGA AAG ATC GTC ATT GTC CGT TAC	486	This study

^a^23S rRNA gene mutation

^b^ *gyr*A gene mutation *Campylobacter jejuni*

^c^ *gyr*A gene mutation *Campylobacter coli*

### Ciprofloxacin resistance

A single point mutation (Thr-86-Ile) in the quinolone resistance-determining region (QRDR) of *gyr*A gene was defined as source of high-level resistance to fluoroquinolones [23]. MAMA–PCR for *C. jejuni* isolates was carried out as described previously [27], for *C. coli* a procedure according to Zirnstein et al. [28] was used. Primer details are given in Table 1.

### Tetracycline resistance

Primers DMT1 and DMT2 (Jena Bioscience GmbH) were used for the detection of the *tet*(O) gene which is strongly associated with tetracycline resistance in *C. jejuni* and *C. coli* as described previously [29]. As a second gene locus associated with tetracycline resistance the presence of *tet*(A) was examined by a previously described PCR assay [22]. An alternative, in-house validated PCR assay was created based on *tet*(A) sequences (GenBank acc. no. JX891463 and JX891464)). Briefly, primers tet-A-1 and tet-A-2 (Table 1; Jena Bioscience GmbH) were used with the following PCR programme: An initial denaturation at 96 °C for 60 s was followed by 35 cycles of denaturation (96 °C for 15 s), annealing (49 °C for 60 s) and extension (72 °C for 30 s). PCR was terminated by final extension at 72 °C for 60 s. The PCR resulted in a 486 bp product.

All PCR products were analyzed by electrophoresis on 1.5 % agarose gels, staining with ethidium bromide and visualization under UV light.

### DNA sequencing

PCR products obtained by *tet*(A) PCRs were sequenced by cycle sequencing with BigDye Terminator v1.1 Cycle Sequencing Kit (Applied Biosystems, Darmstadt, Germany) according to the instructions of the manufacturer. In addition to the amplification primers of the Tet(A)-F/R fragment tet-A-A (5’-AAT TTT CTT CAA ATA AGG-3’) and tet-A-B (5’-GTC ATT CTT ATA TTA AGT GG-3’) were used as sequencing primers. Sequencing products were analyzed with an ABI PRISM 3130 genetic analyzer.

### MALDI-TOF mass spectrometry

Cultured bacteria were suspended in 300 µl of bi-distilled water and mixed with 900 ml of ethanol (Carl Roth GmbH). Further treatment of samples and measurement were described by El-Ashker et al. [30].

## Results

### Identification of bacteria

In total, 58 isolates suspected as *Campylobacter* were recovered from faeces and cloacal swabs of chicken flocks in Kenya. After storage in skimmed milk medium and transportation to Germany only 40 of these isolates could be re-cultivated. Four *C. coli* and 31 *C. jejuni* were identified by mPCR (Table 2). Five other isolates were identified by MALDI-TOF mass spectrometry as members of genera *Bacillus*, *Staphylococcus*, *Ochrobactrum* as well as two *Bordetella* isolates.

**Table 2:** Results of cultivation and multiplex PCR identification of *Campylobacter* isolates

Cultivation	mPCR identification of *Campylobacter*				Total n (%)
	*C. jejuni*	*C. coli*	Not identified	*C. coli/C. jejuni*	
Positive (n)	31	4	5	0	40 (69.0)
Negative (n)	3	14	0	1	18 (31.0)

Eighteen skimmed milk tubes contained *Campylobacter* DNA [14 *C. coli*, 3 *C. jejuni* and one sample harboured both *C. coli* and *C. jejuni* (Table 2)].

### Antimicrobial susceptibility profiles and multidrug resistance

The results of antimicrobial susceptibility testing of *C. jejuni* and *C. coli* isolates and the rate of resistance to seven antimicrobial agents are given in Tables 3 and 4, respectively. The *C. jejuni* isolates showed a high rate of resistance to nalidixic acid, tetracycline and ciprofloxacin with 77.4, 71.0 and 71.0 %, respectively. Low resistance rates were detected for gentamicin and chloramphenicol, both with 25.8 % of the isolates. For the low number of *C. coli* isolates (n = 4) a similar pattern was observed. Only two isolates were susceptible to all tested antimicrobial agents, one isolate was resistant to all tested antibiotics.

**Table 3:**
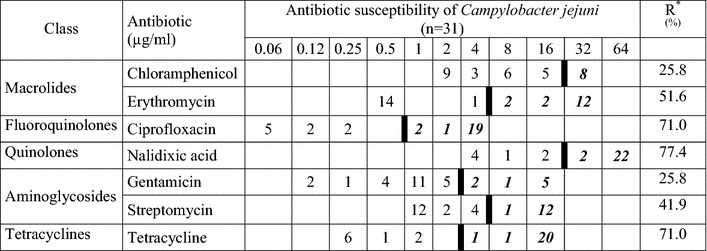
Results of MIC determination and resistance rates of Kenyan *Campylobacter jejuni* isolates

Boldface in italic type indicates the number of resistant isolates. A thick black line indicates the break point between clinically sensitive and resistant strains

R* resistance rate

**Table 4:**
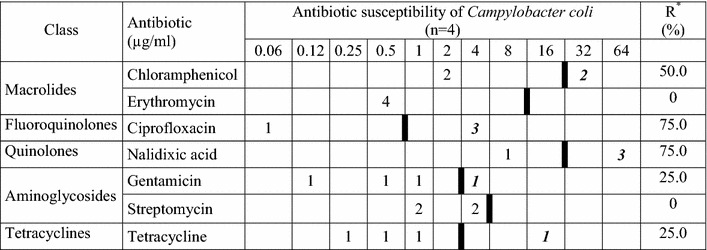
Results of MIC determination and resistance rates of Kenyan *Campylobacter coli* isolates

Boldface in italic type indicates the number of resistant isolates. A thick black line indicates the break point between clinically sensitive and resistant strains

R* resistance rate

The multidrug resistance profiles of 31 *C. jejuni* isolates are shown in Table 5. Multidrug resistance to three or more classes of antibiotics was found in 19 isolates (61.3 %) and was observed in a range between 5.3 and 26.3 %.

**Table 5:** Multidrug resistance profiles of 19 *Campylobacter jejuni* isolates

Antibiotic resistance profile	No. of resistant isolates (%)
TET, CIP, NAL, ERY, GEN, STR, CHL	1 (5.3)
TET, CIP, NAL, ERY, STR, CHL	1 (5.3)
TET, CIP, NAL, ERY, GEN, STR	3 (15.8)
TET, CIP, NAL, GEN, STR, CHL	1 (5.3)
TET, CIP, NAL, ERY, STR	2 (10.5)
TET, CIP, NAL, ERY, CHL	1 (5.3)
TET, CIP, NAL, ERY	2 (10.5)
TET, CIP, NAL, STR	1 (5.3)
TET, CIP, NAL	5 (26.3)
CIP, NAL, ERY, STR, CHL	1 (5.3)
CIP, NAL, STR	1 (5.3)

*TET* tetracycline, *CIP* ciprofloxacin, *NAL* nalidixic acid, *ERY* erythromycin, *GEN* gentamicin, *STR* streptomycin, *CHL* chloramphenicol

### Molecular biological detection of antibiotic resistance determinants

DNA of 35 viable *Campylobacter* isolates and of 18 non-growing samples was investigated by PCR to detect antibiotic resistance. Mismatch amplification mutation assay (MAMA–PCR) was used to characterize a *gyr*A gene mutation associated with ciprofloxacin resistance as well as mutations in 23S rRNA genes as cause of erythromycin resistance. The molecular biological detection of resistance to ciprofloxacin in both *C. coli* and *C. jejuni* was also confirmed by change of amino acid 86 from threonine to isoleucine in the *gyr*A gene. Additional to the ciprofloxacin resistant *Campylobacter* isolates (Tables 3, 4), three *Campylobacter* DNAs were detected harbouring the *gyr*A gene mutation. The results were confirmed by PCR–RFLP according to Vacher et al. [24]. Mutations at positions 2074 and 2075 of the 23S rRNA genes in 13 phenotypically erythromycin-resistant isolates could neither be detected by using PCR–RFLP nor MAMA–PCR.

The *tet*(O) gene which is mainly responsible for tetracycline-resistance was detected by PCR in 12 out of 22 resistant *C. jejuni* isolates (54.5 %) and in all tetracycline resistant *C. coli* isolates. *tet*(O) gene was not detected in DNA extracted from the non-growing samples. Additionally, a newly developed PCR assay was used for the detection of the *tet*(A) gene. *tet*(A) was detected in 28 out of 31 *C. jejuni* (90.3 %) and in all 4 *C. coli* isolates. In 3 out of 14 non-growing samples which harboured *C. coli* DNA, *tet*(A) gene could be found as well as in the one sample where both *C. jejuni* and *C. coli* were detected.

## Discussion

The antimicrobial susceptibility patterns among *Campylobacter* isolates originating from chicken in Kenya were analyzed according to the guidelines of CLSI for *Enterobacteriaceae* which had been guided by previous reports [17, 31–34]. Clinical breakpoints for interpretation of MIC values of *C. jejuni* and *C. coli* from chicken are available [31, 35]. In this study a commercially available broth microdilution assay was used for the determination of MIC values for seven antibiotics. The assay already proved to be suitable in previous investigations [21, 27, 34, 36, 37].

In this study, only 40 out of 58 suspected *Campylobacter* samples could be re-cultivated. The storage conditions (temperature, microaerophilic atmosphere) using skimmed milk medium were possibly not ideal. However, it had been demonstrated that *C. jejuni* can survive up to 14 days at 1 °C or 2.5 days at 20 °C in sterile skimmed milk [38, 39]. Alternative storage of *Campylobacter* cultures using transport medium (for example Amies medium) or cryovials is recommended for future investigations.

In 53 out of 58 collected samples, *Campylobacter* DNA was identified by mPCR assay [26]. The majority of the cultures proved to be *C. jejuni* (88.6 %) which is in agreement with previous studies in chicken [6, 7]. In 18 DNA extracts of non-viable samples, 3 *C. jejuni* (16.7 %), one mixed population of *C. jejuni/C. coli* and 14 *C. coli* were identified. These findings are in agreement with those of a previous study that found a longer viability of *C. jejuni* in comparison to *C. coli* in biological milieu [40]. It may be possible that some *C. coli* isolates had been lost during the shipment period. In summary, *C. jejuni* was identified much more often than *C. coli* (64.2 %) by mPCR investigation. In agreement with other studies, the findings highlighted the usefulness of mPCR as a reliable, sensitive, time and cost saving method for identification of thermophilic *Campylobacter* [26].

The antibiotic susceptibility of 35 *Campylobacter* isolates from Kenyan chicken was investigated using European Committee on Antimicrobial Susceptibility Testing and epidemiological cut-off values (EUCAST–ECOFFS) [16]. A broth microdilution assay was used as a standardized, easy, and reliable method for the determination of MIC of seven antibiotics [17, 31–34]. High resistance rates were obtained for ciprofloxacin, nalidixic acid and tetracycline with more than 70 % which is in agreement with a recent European Food Safety Authority (EFSA) report [41]. These results are in contrast to those of Brooks et al. [5] who reported resistance rates for *Campylobacter* recovered from humans with diarrhoea in Western Kenya for ciprofloxacin, nalidixic acid and tetracycline with 6, 26 and 18 % in 2006, respectively. The general high rates of resistance in the chicken isolates may be caused by availability and uncontrolled use of antibiotics by small farmers [42].

Resistance to chloramphenicol is remarkable with 25.8 % in this investigation. Use of chloramphenicol is banned in animal breeding in Europe for more than 20 years, but still it is often used in many third world countries [43]. It is easy to obtain antibiotics over-the-counter and other unregulated venues and injudicious use promotes the development of resistance to antimicrobial agents. Resistance to gentamicin in the isolates obtained from chicken was low in this study (25.8 %), but *Campylobacter* isolated from broilers and turkeys were totally susceptible to gentamicin [37, 41, 44, 45]. Erythromycin resistance rates found in this study correspond to those of similar studies elsewhere [41, 44, 45].

Multidrug resistance was detected in 61.3 % of the *Campylobacter* isolates. Eleven different combinations were found (Table 5). Frequent, resistance to ciprofloxacin, nalidixic acid and tetracycline was identified (17 out of 19 multidrug resistant isolates) which is in agreement with previous investigation using Vietnamese *Campylobacter* isolates [45]. However, EFSA [41] reported low level of multidrug resistance in *C. jejuni* from broilers of the member states of the EU.

The emerging of antibiotic resistance has been attributed to the overuse and misuse of antimicrobial agents in both the developed and developing world. Antibiotics are widely used as growth supplements in livestock and to prevent infections [46]. The emerging of multidrug resistance may reflect acquisition of different resistance determinants on the same DNA molecule or single determinants, such as multidrug pumps, that specify efflux activity against different antimicrobial agents [47]. The mechanisms of genetic resistance might be chromosomal or plasmid-borne, and represent a combination of endogenous and acquired genes. In general, mechanisms of antibiotic resistance as modification of the antibiotic by aminoglycoside-modifying enzymes (AphA, AadE, Sat), enzymatic inactivation of the antibiotic by β-lactamase and modification of the DNA gyrase target, mutations in 23S rRNA genes were included for aminoglycosides, beta-lactams, fluoroquinolones, macrolides and tetracyclines, respectively [48, 49]. The multidrug efflux pump CmeABC has been involved in the resistance mechanisms of *C. jejuni* and *C. coli* to tetracyclines, fluoroquinolones, macrolides and beta-lactams [49].

Molecular biological methods were used for detection of antibiotic resistance determinants either using DNA isolated from cultures or that of non-cultured bacteria [27, 37]. All isolates of this study which were resistant to ciprofloxacin carried a mutation of the amino acid 86 of the *gyr*A resulting in a change from threonine to isoleucine. This mutation was detected also in 3 DNA samples extracted from skimmed milk. The MAMA–PCR protocol allowed the detection of the *gyr*A mutation and PCR–RFLP was confirming the mutation from (ACA to ATA) of amino acid 86. This result was in agreement with previous reports showing that both methods are simple, reliable, rapid tools that can be used as screening methods [27, 37]. In *Campylobacter*, resistance to erythromycin is chromosomally encoded by an alteration of the 23S rRNA gene. High level resistance to erythromycin is caused by mutations at position 2074 and/or 2075 of the domain V of this gene. In this study the mutations were neither detected by MAMA–PCR nor by PCR–RFLP.

The *tet*(O) gene is known to be responsible for tetracycline resistance in *Campylobacter* isolates [29]. In this study, only 54.5 % of the tetracycline resistant isolates harboured the *tet*(O) gene. The *tet*(A) gene also plays role in resistance to tetracycline [22]. The efflux gene *tet*(A) is coding for an approximately 46 kDa membrane-bound efflux protein for membrane-associated proteins and is involved in the export of tetracycline from the cell [50]. In this study, using the recommended primers for *tet*(A) amplification [18] PCR products of 696 bp instead of 888 bp were obtained. DNA sequencing of amplicons and database search resulted in 99.0 % homology to a partial putative integral membrane protein and a putative periplasmic protein. Hence, a new PCR assay based on *tet*(A) gene sequences for *C. jejuni* (acc. no. JX891464) and *C. coli* (acc. no. JX891463) was developed. Parameters such as limit of detection, limit of quantification, PCR efficiency and specificity were considered during an in-house validation process. Amplicon length was 486 and the amplicons were sequenced to confirm the identity. The *tet*(A) gene was much more frequently identified in the Kenyan *Campylobacter* isolates than *tet*(O) (35 vs 13).

To the best of our knowledge this is the first report on the status of antibiotic susceptibility of thermophilic *Campylobacter* from chicken in Kenya. High level of resistance to ciprofloxacin, erythromycin and nalidixic acid as well as multidrug resistance was detected previously in Kenya. In Kenya, this problem is reported to be caused by the increasing rate of unregulated over-the-counter sale without prescriptions of these antibiotics, mainly to humans self-treatment of suspected infections and to a lesser extent for use in animals [51]. These findings also demonstrate the potential for antibiotic-resistant bacteria to spread through the food chain from animals treated with antibiotics for humans. Such misuse and overuse may have resulted in the selection of resistant mutants or acquisition of antibiotic resistance genes from other organisms through the process of genetic exchange.

It is recommendable that a long-term local surveillance programme is adopted for monitoring changes in resistance among *Campylobacter* isolates. Efforts to establish at least a National Laboratory with facilities for performing phenotyping and genotyping methods is highly recommended. Emphasis should be given on educational advertising to reduce the input of antibiotics in animal breeding to minimize the potential hazard for humans.
